# A mixed methods study investigating factors affecting adherence to *Plasmodium vivax* malaria primaquine radical cure regimens among migrants along the Myanmar-Thailand border

**DOI:** 10.1371/journal.pgph.0003615

**Published:** 2025-01-16

**Authors:** April T. Ansari, Ko Ko Aung, Htun Htun Win, Candy Beau, Be Nu, Nay Lin Soe, Klay Htoo, Thida San, Tha Gay Wah, Arunrot Keereevijit, Aung Pyae Phyo, Kesinee Chotivanich, Nicholas J. White, François Nosten, Ahmar H. Hashmi, Cindy S. Chu

**Affiliations:** 1 College of Health Sciences, The University of Texas at El Paso, El Paso, TX, United States of America; 2 Shoklo Malaria Research Unit, Mahidol–Oxford Tropical Medicine Research Unit, Faculty of Tropical Medicine, Mahidol University, Mae Sot, Thailand; 3 Mahidol–Oxford Tropical Medicine Research Unit, Faculty of Tropical Medicine, Mahidol University, Bangkok, Thailand; 4 Department of Clinical Tropical Medicine, Faculty of Tropical Medicine, Mahidol University, Bangkok, Thailand; 5 Centre for Tropical Medicine and Global Health, Nuffield Department of Medicine, University of Oxford, Oxford, United Kingdom; 6 Department of Nutrition Sciences and Health Behavior, School of Health Professions, University of Texas Medical Branch, Galveston, Texas, United States of America; Menzies School of Health Research, AUSTRALIA

## Abstract

**Background:**

The countries within the Greater Mekong Region of Southeast Asia have pledged to eliminate malaria by 2030. Elimination of *Plasmodium vivax* malaria is challenging as it requires radical cure to prevent relapse. Understanding and facilitating adherence to primaquine radical cure regimens is necessary for malaria elimination.

**Methods:**

A convergent parallel mixed methods study was conducted to investigate the barriers to and facilitators for completing primaquine treatment of *P*. *vivax* infection among mobile migrant communities on the Myanmar-Thailand border. Quantative data were derived from routine malaria consultations. Qualitative data, informed by the social cognitive theory and health belief model, were collected through in-depth interviews with patients and focus group discussions with local health providers and community leaders.

**Results:**

Of 729 adult patients with primaquine treatment outcomes, 45% did not complete the follow-up of 28 days and were assumed to be non-adherent to primaquine treatment. Patients of Karen ethnicity (OR 1.7, 95% CI 1.2–2.3; p = 0.001) or having a previous episode of malaria from any species (OR 1.6, 95% CI 1.1–2.3; p = 0.007) were more likely to report completing the 14-day primaquine radical cure regimen. Five focus group discussions with front-line healthcare workers and community members and 16 in-depth interviews with patients who were prescribed *P*. *vivax* radical cure were conducted. Key themes related to the social cognitive theory included behavioral factors where work outweighed the choice to complete treatment; environmental factors where access to care determined primaquine treatment completion; and cognitive factors having a positive but limited influence on treatment completion. According to the health belief model, prioritizaton of work reduced seeking diagnosis and completing treatment, and often outweighed facilitating factors such as malaria literacy, health education, and social norms; and affected the perceived susceptibility and severity of *P*. *vivax* infections.

**Discussion:**

Work and productivity were identified as primary behavioral factors affecting adherence to primaquine radical cure and follow up in a migrant population. Community support and cultural cues may overcome these barriers. Understanding the rationale of patient adherence to primaquine may help guide programming for *P*. *vivax* elimination among migrant populations in resource-constrained settings.

## Introduction

Malaria elimination activities by countries in the Greater Mekong Region (GMR) of Southeast Asia have significantly reduced the incidence of *Plasmodium falciparum* infections so that *Plasmodium vivax* is now the most common human malaria in the region [1]. Primaquine is required for the radical cure of *P*. *vivax* to eliminate hypnozoites from the liver and prevent relapses of vivax malaria. Without radical cure, *P*. *vivax* infections can recur weeks to months later [2, 3]. The full course of primaquine in the GMR typically has been 14 days, except for glucose-6-phosphate dehydrogenase deficient (G6PD-deficient) individuals who are prescribed primaquine weekly for 8 weeks [4]. Full adherence to the primaquine regimen optimizes treatment efficacy and supports the radical cure of *P*. *vivax* in the final stages of malaria elimination in the GMR [1, 4–6].

Though less fatal than *P*. *falciparum*, frequent *P*. *vivax* infections and relapses increase medical costs, decrease productivity time [7], and increase risk for re-hospitalization [8]. In 2017, the economic burden of *P*. *vivax* in Thailand was estimated to be over $300,000 USD annually due to provider, direct household, and indirect household costs [9].

Adherence to the 14-day and 8-week regimens of primaquine is low, especially among migrant, border, and ethnic minority populations across Asia. Patients receiving unsupervised treatment have lower rates of adherence compared to those being directly observed [5, 6, 10–13] and experience more *P*. *vivax* recurrences [5, 6, 10–14]. Although many studies have measured adherence rates through quantitative methods and laboratory confirmation, they provide limited psychosocial explanations of the root causes of poor adherence in these difficult-to-reach populations [1, 15–17]. Informed consent, direct observations by research staff, and the use of diagnostic tests contribute to higher adherence than may occur in real-world settings [16]. Other quantitative measures of adherence may be vulnerable to recall bias by using self-reports and pill counts [15, 16]. Finally, short follow-up durations limit the accuracy of *P*. *vivax* recurrence estimates [1].

For migrant populations access to malaria diagnosis and treatment may be constrained by limited or costly transportation, uncertain security during travel, weather conditions, and/or unconventional working hours [18]. Fear of productivity loss may incentivize patients to adhere to treatment [5, 7]. Community health workers play a significant role in building trust, which aids patient primaquine adherence [19]. Malaria literacy, higher education levels, and higher household incomes also increase adherence [5, 15, 20], although these beneficial factors are likely to be limited in migrant populations.

Only a few malaria studies [15, 20–22] have used social and structural determinants to prospectively assess adherence, and even fewer have attempted this with primaquine adherence. Common recommendations for improving primaquine adherence include 1) supervised treatment, 2) a shorter radical cure treatment regimen, 3) ongoing evaluation of antimalarial drug efficacy and resistance, 4) health education and monitoring through information and communication technology, and 5) mandatory G6PD testing to mitigate the adverse effects of hemolysis [3, 9, 23–25]. However other factors, not measured in quantitative studies, may be equally or more important.

The aim of this study was to use mixed methods, guided by social behavioral theories, to provide a comprehensive understanding of the factors related to primaquine adherence in mobile, migrant populations along the Myanmar-Thailand border.

## Methods

### Study design

**Fig 1:**
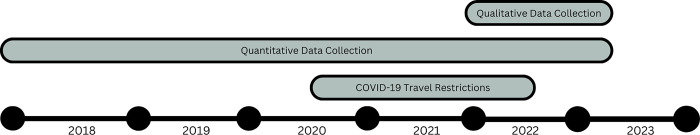
Timeline of the quantitative and qualitative studies. The timing of the COVID-19 pandemic travel restrictions is included as it impacted patient’s access to health care and follow-up.

This study employed a convergent parallel mixed methods design, in which quantitative and qualitative data are collected simultaneously over the same time period and subsequently analyzed [26]. The quantitative data were collected between 01/01/2018 and 30/04/2023 (data accessed 01/05/2023) during routine malaria consultations at SMRU outpatient clinics (Fig 1). Qualitative data were collected from 01/12/2021 to 31/03/2023 through in-depth interviews (IDIs) and focus group discussions (FGDs). Anonymized clinical data were used to identify other factors affecting patient adherence to primaquine treatment, such as demographics, clinical characteristics, disease severity, and treatment supervision. Quantitative data were supplemented by qualitative data, which corroborated and expanded on understanding of primaquine treatment adherence in the migrant communities along the border.

### Study setting

This study was conducted by the Shoklo Malaria Research Unit (SMRU). From 1986 to 2017, SMRU provided health programming for refugees (“persons fleeing violence”) in refugee camps in Thailand [27]. In 1998, SMRU established clinics to serve economic migrant communities along the Thailand-Myanmar border as well [27]. Although SMRU operations in refugee camps have closed, they are still ongoing in migrant communities. These migrant communities are comprised of economic migrants mainly of Burman and Karen ethnicities, who take up temporary residence on either side of the Thailand-Myanmar border. Many of these migrants seek work and essential services (i.e., health care) within Thailand [28].

In this study conducted from 2018 to 2023, study participants were from the migrant communities presenting for care at SMRU clinics located in Thailand. “Migrants” are considered to be “voluntary immigrants” from Myanmar into Thailand seeking economic benefits. Although Thailand has a system for registering migrants for public health services, many of the migrants presenting to SMRU for health care have no formal migrant documents indicating registration [29, 30]. Migrants have variable lengths of stay on the Thailand-Myanmar border. Depending on the seasonality and work availability, migrants commonly work as daily wage laborers and are usually mobile within the border region [31–33].

In the outpatient clinics, malaria diagnosis, G6PD testing, anemia assessment, and blood stage malaria treatment are provided free of charge. Primaquine regimens to interrupt *P*. *falciparum* transmission (single dose primaquine) and for radical cure against *P*. *vivax* hypnozoites (primaquine 0.5 mg/kg/day for 14 days) have been included as part of routine outpatient clinical care since 2014 and 2017, respectively [34]. During routine clinical treatment of *P*. *vivax* malaria, supervised primaquine is not required. The clinical decision to supervise primaquine doses takes into account various patient factors such as age, presence of anemia, distance from the clinic, and the healthcare worker’s perception of a patient’s understanding of how to take primaquine. For example, some patients are prescribed a full course of primaquine unsupervised and others may receive a partial course of primaquine with a follow-up visits before the remaining tablets are prescribed. Additionally, if a health care provider is concerned about side effects (e.g., anemia or potential hemolyis), they request additional follow-up in the first week of treatment to review the patient’s clinical status. Supervised treatment for the weekly primaquine regimen in G6PD-deficient patients is required.

### Quantitative data: Collection and analysis

The quantitative data were collected from the routine clinical consultations of malaria patients in two outpatient clinics from 01/01/2018 to 30/04/2023. Health care access in the migrant population was limited during the COVID-19 pandemic because of border travel restrictions from March 2020 to May 2022 (Fig 1). Baseline demographics, medical history, clinical information, and laboratory results (e.g., malaria smear, G6PD testing, complete blood count) were recorded. To document whether a primaquine dose was supervised, the treatment start date, regimen (e.g., weekly or daily), and day of dose supervision were recorded. To document primaquine adherence, the health care provider would confirm whether the patient completed prescribed regimen verbally with the patient by a follow up visit, mobile phone contact, or home visit after day 14 and by day 28. Patients who did not follow up in person at the required day 28 visit who also could not be contacted by mobile phone or home visit since day 14 were assumed to be non-adherent to primaquine. All visit for acute malaria illness were detected passively. The data were de-identified so that the participants in the qualitative study could not be linked to their clinical data.

Clinical and laboratory data were recorded to a paper case report form and entered manually into MACRO. A unique patient identifier was assigned to each patient along with the date of the current malaria infection. Only adults ≥18 years old presenting for their first episode of *P*. *vivax* malaria were included in the overall analysis. Univariable logistic regression was used to determine the differences between those who were or were not adherent to primaquine for the following variables: sex, ethnicity, duration of stay in Thailand (a measure for recent migration from Myanmar), a history of any malaria, duration of fever (an indicator of severity), hematocrit, and being treated during COVID-19 travel restrictions. Multivariable logistic regression was used to compare the relationship between primaquine adherence and *P*. *vivax* recurrences, and included the above patient characteristics as co-variates. Ordered logistic regression was used to determine which patient characteristics were associated with the total number of supervised doses in the first week of daily dosing (reflecting the health care provider’s subjective decision to request early follow up visits). Weekly dosing in G6PD-deficient patients was excluded from this analysis. Since G6PD-deficient patients are predominantly male, sex was also removed from this analysis.

### Qualitative data: Theoretical frameworks

**Fig 2:**
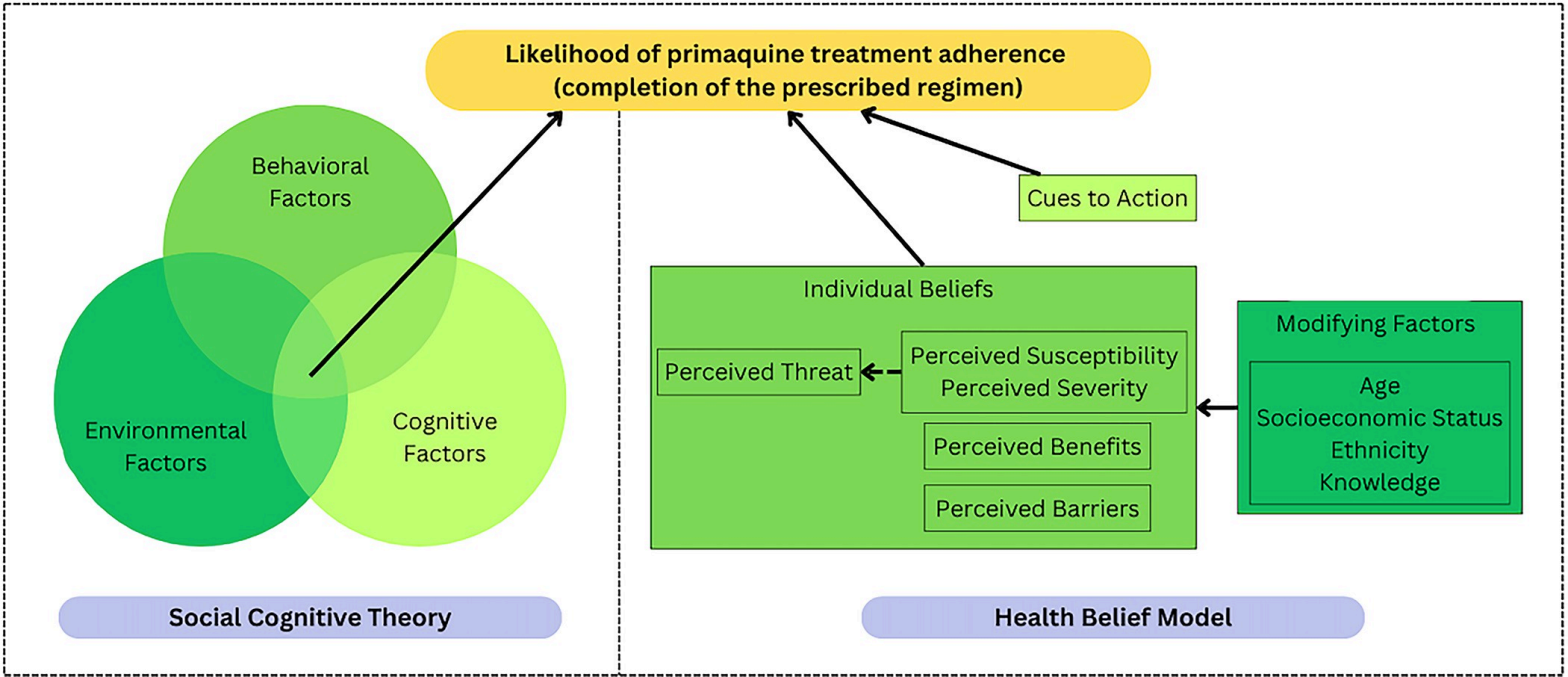
Theoretical frameworks for the Social Cognitive Theory (SCT) and Health Belief Model (HBM). Green shading denotes different categories within each behavioral theory.

This study was underpinned by the theoretical frameworks of the Social Cognitive Theory (SCT) and Health Belief Model (HBM) to describe why participants may or may not complete their primaquine treatment (Fig 2). Built upon the research of Miller and Dollard, the SCT claims that individuals and environments influence each other by reciprocal determinism, ultimately shaping human actions through environmental, cognitive, and behavioral factors [35]. The HBM was developed by the U.S. Public Health Service in the 1950s to explore how modifying factors, such as social determinants of health, influence individual beliefs that determine human behavior [36]. Certain factors may overlap between the SCT and HBM, such as concepts related to self-efficacy and outcome expectations. The scientific rationale for using both theories allows for environmental considerations by using the SCT, while the HBM allows a more patient-centered understanding. Presenting findings from both models may provide evidence towards initial steps to improve adherence to a primaquine regimen that supports decisions made by researchers, public health practitioners, and policymakers.

The ontological grounding for the qualitative methods was rooted in a constructivist paradigm, which assumes that there is more than one reality based on the participants’ experiences when studying primaquine adherence [37–39]. Study design and analysis followed a subjectivist deductive approach [39, 40], which allows use of subjective processes in understanding participant responses as guided by the SCT and HBM constructs [41]. The SCT and HBM theoretical models and conceptual premises together provide a more comprehensive understanding of primaquine adherence for *P*. *vivax* treatment.

### Qualitative data: Collection and analysis

Patients who were diagnosed with *P*. *vivax* infection were screened for eligibility by a health care provider within one month of the last day of their primaquine regimen. They were enrolled if they were willing and able to be interviewed, ≥18 years old, and provided written informed consent. Health care professionals experienced in *P*. *vivax* treatment and non-medical community leaders were selected for FGDs because of their engagement with the community at large, thereby representing malaria patients who did not seek treatment at a SMRU clinic or were lost to follow-up. Purposive sampling resulted in groups of four to six participants with similar backgrounds to allow for deeper discussions. Data saturation was reached when IDIs and FGDs no longer provided new information or themes as determined by research team consensus (KKA, CC, AH, AA). All study participants were adults (≥18 years) who communicated in either Burmese or Karen, were part of migrant communities served by SMRU, and provided written informed consent for participation.

SMRU research team members comprised a clinical research doctor (CC), mixed methods expert (AH), research assistant (AA), and a non-medical local facilitator (KKA). KKA has previous experience conducting IDIs and FGDs, was trained on specific research questions, and received instruction from CC and AH on interviewing, discussion, and notetaking techniques. IDIs and FGDs were led by KKA who was not responsible for any provision of clinical care.

Semi-structured IDI and FGD guides were developed using the constructs from the SCT and HBM. All IDIs and FGDs were conducted in person with appropriate COVID-19 precautions being taken. IDIs were conducted at the SMRU clinics and FGDs were conducted in a meeting place agreed upon by the focus group participants for ease of access. Following IDIs and FGDs, research team members debriefed to identify key themes, discuss the progress of the study, and determine whether data saturation was reached. IDIs and FGDs were audio-recorded, translated, and transcribed verbatim to English, and imported into NVivo software to analyze the main thematic content.

**Fig 3:**
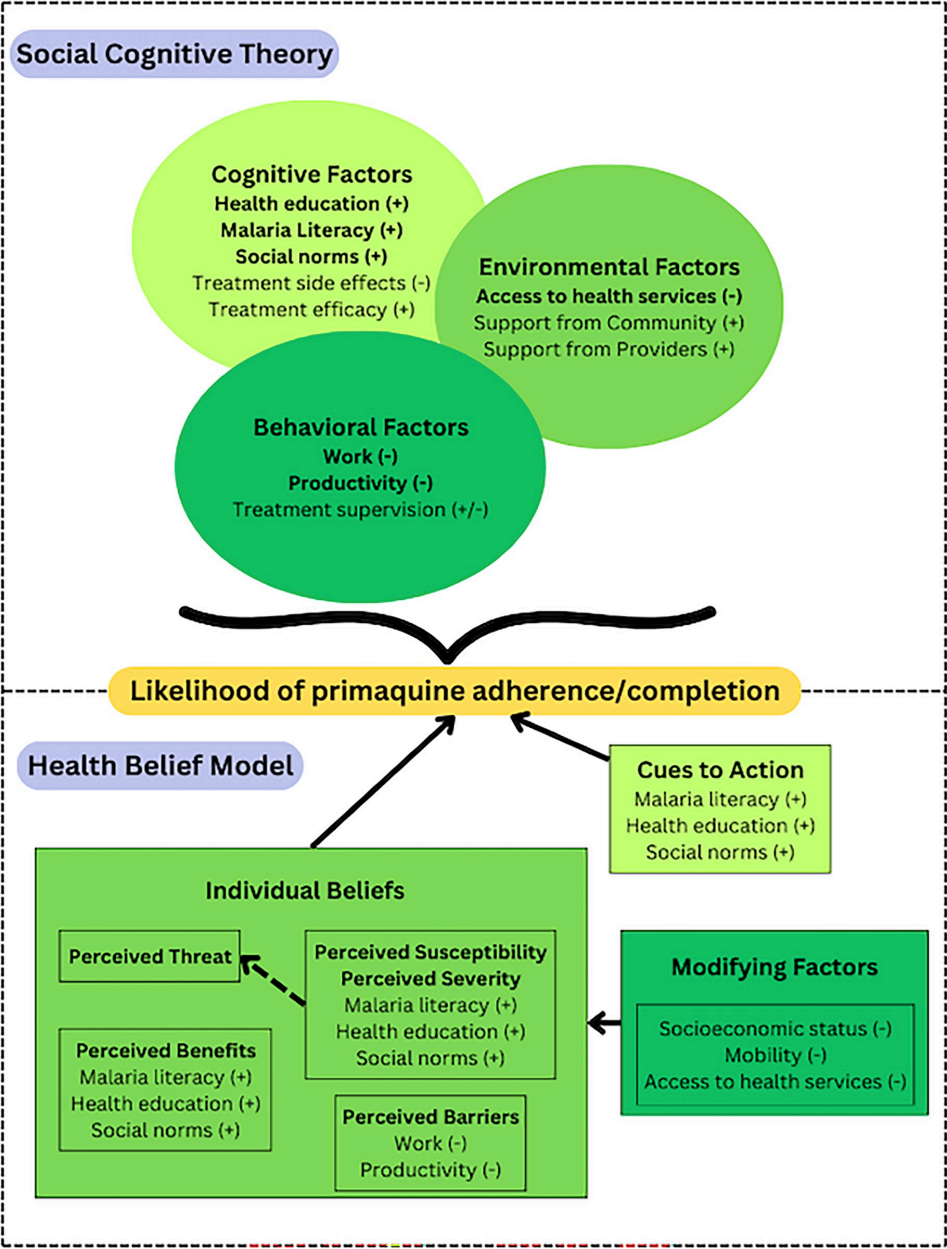
Social Cognitive Theory (SCT) and Health Belief Model (HBM) populated with factors determined through qualitative analysis. (+) and (-) indicate factors with a positive or negative influence on primaquine adherence, respectively. Green shading denotes different categories within each behavioral theory.

Deductive coding was performed by two research team members (AA, AH) using SCT and HBM constructs to guide preliminary analysis. Coding comparisons were conducted to assess inter-observer reliability and examine discrepancies between research team members. Discrepancies in coding were then addressed, the codebook was finalized based on theoretical frameworks (Fig 3), and AA reviewed all transcripts and coded accordingly. Thematic analysis was then performed (AA, AH) in NVivo based on SCT and HBM constructs. Feedback was provided by CC and KKA throughout the process, allowing for triangulation and corroboration to enhance the validity of key findings.

### Ethics statement

Ethics approval was given by the Ethics Committee at the Faculty of Tropical Medicine, Mahidol University (TMEC 21–007 and 23–036) and the Oxford Tropical Research Ethics Committee (OXTREC 510–21 and 532–23). Additional information regarding the ethical, cultural, and scientific considerations specific to inclusivity in global research is included in the Supporting Information (S1 Checklist).

## Results

### Quantitative analysis

**Fig 4:**
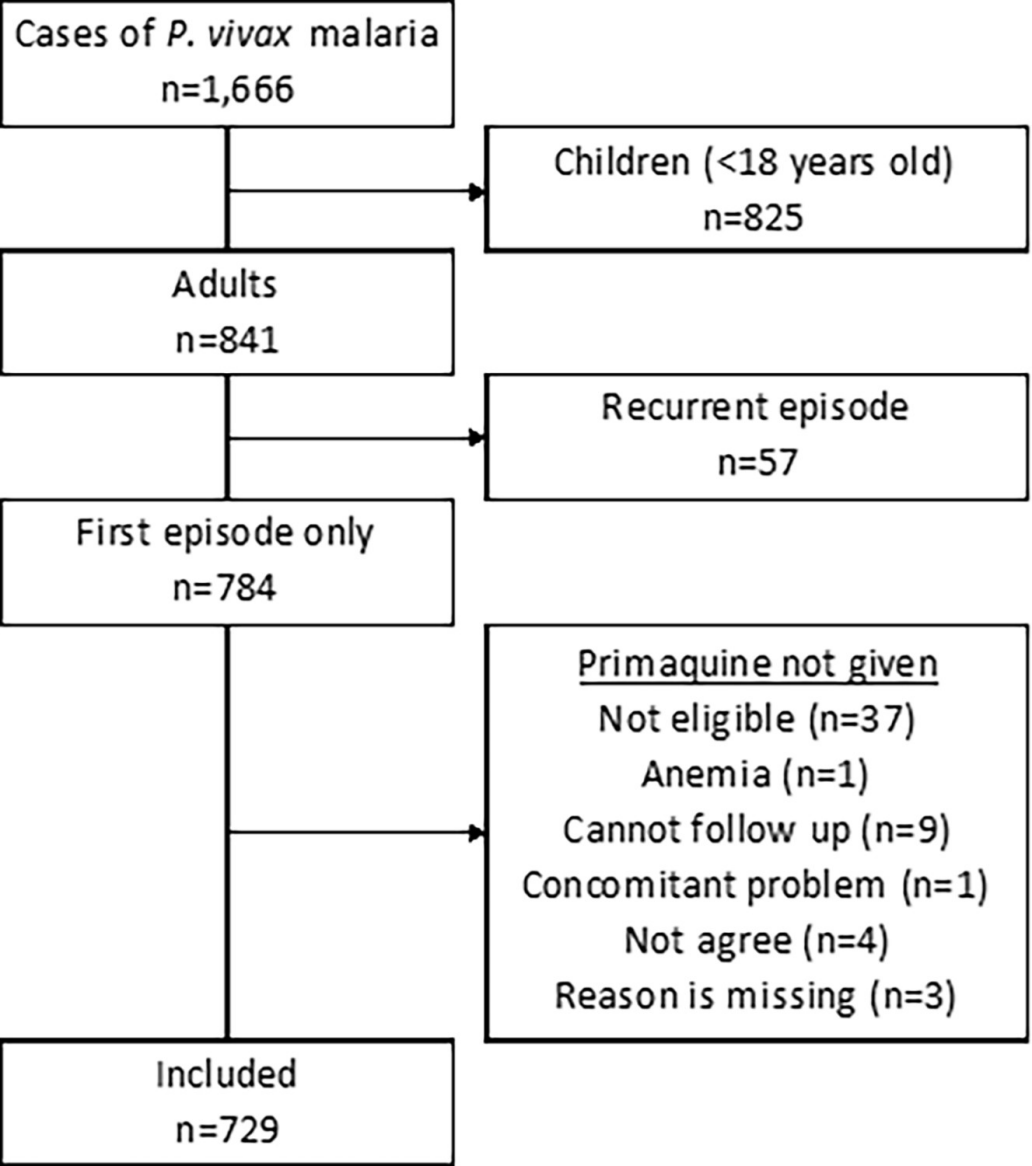
Trial diagram for the quantitative study. The participants in the qualitative study were also patients. Thus, their clinical data are included in the quantitative analysis.

From January 2018 to April 2023, there were 729 patients ≥18 years old diagnosed with *P*. *vivax* malaria who were prescribed primaquine for radical cure (Fig 4). The IDI participants were part of this cohort thus their de-identified data were included the analysis. Of the *P*. *vivax* patients receiving primaquine, 329/729 (45%) were not adherent to primaquine treatment, either by self-report over the phone (25/329; 8%) or they missed the day 28 clinic visit (306/329; 92%). The remaining 400 patients (55%) were adherent to their primaquine treatment; 184 of them (37%) missed the day 28 visit but self-reported over the phone that they had completed primaquine treatment.

In the univariable logistic analysis, there were two characteristics associated with higher rates of primaquine completion: being of Karen ethnicity, odds ratio (OR) 1.7 (95% CI 1.2–2.4; p = 0.001) and having a previous episode of any malaria, OR 1.6 (95% CI 1.1–2.3; p = 0.008; Table 1). Age, sex, the duration living in the area (a proxy for migratory status), and being treated during COVID-19 travel restrictions were no different between patients who were adherent to primaquine or not (Table 1). When all of these factors were included in the multivariable logistic regression, only being of Karen ethnicity was associated with higher odds of primaquine adherence, OR 1.5 (95% CI 1.1–2.1); p = 0.01.

**Table 1:** Patient characteristics in the quantitative study. The univariable generalized linear model with binomial family and logit link analysis is shown in this table. When all variables were included, Karen ethnicity and a history of any malaria continued to have higher odds of completing primaquine (PQ). IQR: Interquartile Range.

	PQ given, non-adherent	PQ given, adherent	Odds ratio (95% CI)	p-value
	n = 329	n = 400		
Male, n (%)	229^1^ (69)	266^2^(66)	0.9 (0.6–1.2)	0.4
Age, median years (range)	26^1^ (18–74)	28^1^ (18–77)	1.0 (0.99–1.02)	0.2
Number of years living in Thailand, median years (IQR)	2^3^ (0.3–8)	3^4^ (1–10)	1.02 (1.0–1.03)	0.05
Ethnicity, n (%)				
Karen	201 (61)	291 (73)	1.7 (1.2–2.4)	0.001
Burman	121 (37)	102 (25)	comparator	
Other	7 (2)	7 (2)	1.2 (0.4–3.5)	0.8
Medical history with any malaria, n (%)	65^1^ (20)	113^5^ (28)	1.6 (1.1–2.3)	0.008
Fever duration, median days (IQR)	3^1^ (2–4)	3 (2–4)	1.0 (0.9–1.0)	0.5
Hematocrit (%), mean (range)	40^5^ (25–54)	40^6^ (21–65)	0.9 (0.9–1.0)	0.4
Treated during COVID-19 travel restrictions (Mar 2020-Mar 2022), n (%)	36 (11%)	58 (14%)	1.4 (0.9–2.2)	0.2

Univariable logistic regression results are shown in this table.

Missing data

^1^n = 1

^2^n = 2

^3^n = 3

^4^n = 4

^5^n = 5

^6^n = 9

The number of subsequent *P*. *vivax* recurrences (passive case detection during the course of the study) was similar for patients adherent to primaquine treatment (23/400; 6%) and those who were not (19/329; 6%); OR 1.0 (95% CI 0.5–2.0; p = 1.0).

All patients received supervised primaquine on the day of diagnosis. Approximately 10% of patients receiving daily primaquine doses had at least one follow up visit in the first week of treatment. Patients with a previous history of malaria were more likely to have at least one scheduled follow up in the first week, OR 1.7 (95% CI 1.3-2-3; p = 0.001). During COVID travel restrictions from March 2020 to 2022, scheduled follow up visits in the first week were less likely, OR 0.47 (95% CI 0.3–0.9; p = 0.02).

### Qualitative analysis

Out of five FGD (total of 32 participants), four FGD were conducted with local frontline health workers providing malaria diagnosis and treatment, and one FGD consisted of community members. A total of 16 IDIs were conducted with patients who had completed primaquine treatment in the last month. Factors influencing primaquine adherence overlapped between the SCT and HBM, but the relationships between factors differed (Fig 3). A sample of quotes according to the thematic analysis for both behavioral models are included in S1 Table.

### Results according to the Social Cognitive Theory

The results of the thematic analysis guided by SCT suggest that behavioral factors most significantly impacted patients’ likelihood of primaquine completion for the radical cure of *P*. *vivax*. The results demonstrate that the most salient factors determining primaquine adherence were related to the need to work. Migrant patients perform predominantly agricultural work as available, fluctuating throughout the year according to farming seasons. Often, employment is irregular, with migrant workers receiving work on a day-to-day basis. The daily minimum wage for migrants in Thailand is between 328–354 THB (USD $9.01–9.72). Men work predominantly outside of the home, however there were no significant differences in the proportion of men completing primaquine treatment compared to women who work predominantly inside the home (Table 1). Although the need to work and earn a daily wage influenced all constructs (behavioral, environmental, and cognitive factors), it was most important as a behavioral factor. The need to work limited access to health services, another important factor related to completing primaquine treatment. The results are structured to illustrate the most important factors related to primaquine adherence: behavioral factors, environmental factors, and cognitive factors.

### Behavioral factors: The need to work outweighs the decision to complete treatment

The important subthemes among behavioral factors were work, productivity, and drug administration (i.e., supervised versus unsupervised treatment). Health workers identified issues with clinical symptoms, which if resolved quickly placed patients at risk for not following up and not completing primaquine treatment. They explained that patients would feel ‘healthy’ enough to stop treatment and desire to return to work. Conversely, if patients experienced adverse effects (e.g., dizziness or nausea) they might stop treatment so they could return to work. Although health workers explained to patients why they must complete treatment to avoid recurrent infections, patients’ immediate concerns were to prevent any productivity loss attributed to primaquine side effects. In addition, needing to work represented a competing priority whereby patients often reported difficulties remembering to take their primaquine treatment or taking their medicines with them to work. According to a community leader,

“Most migrant workers say they have dizziness and cannot work if they take malaria drugs. Their main purpose is to earn money to repay their debts or to build new houses for themselves. The main reason is a money issue.”—Community Leader, FGD

As one patient noted,

“I forgot to take malaria drugs for two days when I was breeding cattle to earn money. I forgot to bring drugs with me.”—Patient, IDI

Consequently, health workers advocated for supervising primaquine administration to verify treatment completion and learn about patient needs and treatment side effects that otherwise may not notice or report without daily or regular follow-up visits. However, many noted how difficult this was in practice:

“It is especially difficult during the seasonal working period, after [the patients] finish work they are very tired and the drug makes them even more tired. But we explained to them how important it is to take the complete dose, that they cannot just quit in the middle of the treatment. For [health workers managing malaria posts in Kayah State, Myanmar], it’s very difficult to ask the patients to come back and take the medicine every day for 14 days. It is not convenient for the patients to come back either.”—Health care worker, FGD

### Environmental factors: Access to care dictates primaquine treatment completion

The important subthemes among environmental factors were access to health care, support from the community, and support from providers. The prioritization of work and productivity determined access to health care for diagnosis, treatment, and follow-up. The activity spaces (i.e., geographical areas where people move around to conduct their daily activities) [42] of mobile migrant populations were built around their work sites, typically in remote areas. Traveling far in distance, securing transportation, and scheduling clinic visits around long and variable working hours served as environmental barriers for patients in accessing health services. According to a health worker,

“Even though some [patients] belong to this village, they don’t live in it—they live on their farm or in the field and it is not near their village so it’s hard for them to come back and take medication.”—Health care worker, FGD

As primaquine requires a long treatment course, providers preferred patients visit the clinic daily to supervise drug administration and ensure completion. Migrant populations were less likely to agree to scheduled visits due to environmental barriers; therefore, providers offered an alternative option for these patients to travel home with their pills and administer their regimen without medical supervision. The quantitative analysis did not show a difference in passively detected *P*. *vivax* recurrences between adherent and non-adherent to primaquine treatment.

Concern over work and productivity determined access to health services; however, patients and health workers discussed support from the community and providers in helping overcome environmental barriers. Many patients noted that a family member or friend had driven them to a clinic for malaria diagnosis and treatment.

“My aunt has many friends at the clinics. So she made phone calls and they picked me up to the clinic….I also have many friends. I called the staff to pick me up and came to the clinic.”—Patient, IDI

However, health workers showed concern for low rates of patients returning for follow up visits.

“We give medicine for malaria. We give medicine for anemia if they need. We also give soap and instant noodles and tell them to come back for follow up. For some it is difficult to come back because of their work and children. They usually do not come back for follow up at 1 or 2 weeks.”—Health care worker, FGD

As a result, health workers recommended that providers should devise better strategies for locating and maintaining contact with patients to ensure primaquine treatment completion that can help overcome issues including geographical distance and the mobility of the migrant population.

### Cognitive factors have a positive, but limited, influence on treatment completion

Given the priortization of work and productivity, and the environmental barriers to healthcare access that arise because of the need to work, cognitive factors had a limited influence on the likelihood of completing primaquine treatment. Cognitive factors broadly covered themes such as health education, malaria literacy, social and community norms, treatment side effects, and confidence in treatment efficacy. Both health workers and patients confirmed that providers reinforce the importance of treatment completion through comprehensive health education. In this setting, patients exhibited high malaria literacy (i.e, awareness of symptoms, prevention measures, and the need for diagnosis and treatment). Most patients interviewed demonstrated proficiency in pertinent health information related to primaquine treatment completion. Despite the positive influences malaria literacy and health education had, other cognitive factors interfered with patients turning their understanding of its importance into treatment completion. For instance, patients reported difficulties in bringing their pills to work, remembering to take their pills, and adverse side effects that disrupted their work.

Health workers reported that treatment side effects, such as dizziness and weakness, were the leading cognitive factor limiting full primaquine adherence, especially among migrant populations who consider work a priority. Though they counseled patients on the adverse effects of primaquine (e.g., taking tablets after a meal prevents abdominal pain), the willingness of patients to complete treatment was undermined by perceived or actual adverse effects that affected their occupations as seasonal farm workers. Since the type of work migrants perform requires intense physical labor, negative side effects prevented patients from prioritizing early detection and treatment or treatment completion. As one health worker stated,

“Even for us, it’s really hard to urge and persuade them to come back once a day for treatment. It is especially more difficult during the seasonal working period. After they finish work, they are very tired, and the drug makes it more difficult for them.”—health care worker, FGD

For this reason, health workers focused their communication with patients to prepare them for the potential side effects of primaquine and encouraging them to seek care if needed. Meanwhile, patients highlighted the role of social and community norms in motivating patients to seek care and building their confidence in treatment efficacy. According to a patient,

“I see everybody coming to the clinic when they are unwell. Malaria will not be cured if we do not come to the clinic. My friends came to the clinic when they got malaria. Malaria is not cured at home.”—Patient, IDI

Confidence in treatment efficacy was also related to trust in providers. Many patients sought malaria care at a SMRU clinic after witnessing a family member, friend, or neighbor treated previously for malaria and/or being advised by one of them to seek care. Similarly, patients’ social circles supported primaquine adherence in reminding patients to take their pills every day. Patients were motivated by social and community norms to seek care and were further driven to complete treatment for the purpose of feeling better and avoiding recurrent infections, especially since feeling ill limited their ability to work.

“I am afraid of suffering malaria again. I don’t like to be unwell. I want to be cured completely. I cannot work if I am unhealthy. So I took it regularly until the treatment was finished.”—Patient, IDI

This is corroborated by the quantitative findings that show patients with any previous malaria infection were more likely to attend a scheduled follow-up within the first week of primaquine treatment and complete primaquine treatment (Table 1). The quantitative results also support the role of the community (health care providers are predominantly of Karen ethnicity and Karen patients were more likely to complete primaquine treatment) in seeking and adhering to malaria treatment.

### Health Belief Model

While the SCT contextualized the interactions between behavioral, environmental, and cognitive factors, the HBM offered deeper insights into how cognitive factors such as perceptions and individual beliefs affected behaviors related to primaquine adherence. Given the similarities between the two theories, environmental factors from the SCT aligned with modifying factors in the HBM, such as socioeconomic status, mobility, work, productivity, and access to health services (Fig 3). Migrants’ prioritization of work increased perceived barriers to seeking diagnosis or completing treatment. Prioritizing work served as both modifying factors and perceived barriers in seeking clinics to confirm their diagnosis, completing the regimen without compromising their availability for work, and perceived side effects that would effect their capacity for work.

On the other hand, malaria literacy, health education, and social norms raised the perceived susceptibility and severity of *P*. *vivax* infection as patients were aware of the consequences of not completing primaquine treatment. Patients who understood and feared recurrent infections reported full primaquine adherence. In combination with modifying factors, perceived susceptibility and severity directly shaped perceived threat and harm, which were further informed by strategies that promote primaquine adherence (i.e., “cues to action”). For primaquine adherence, cues to action overlapped with factors that increased perceived susceptibility and severity, including malaria literacy, health education, and social norms. The clinical teams are predominantly of Karen ethnicity, and this may have contributed to increasing the social cues to action in patients who are of the same ethnicity. This could explain why patients of Karen ethnicity were more likely to complete primaquine treatment as shown in the quantitative analysis (Table 1). In addition, cues to action and perceived threat/harm influenced perceived benefits of treatment, such as the desire to feel better, return to work, and avoid recurrent infections.

A comment from one of the patients helps illustrate the balance between perceived barriers, perceived benefits, and support needed in completing treatment:

“I told the health workers that I had to work. I had limited time to come to the clinic and it is very far. Fortunately, [the health worker and I] made a plan to take malaria treatment at ** village with one of health workers living there. He asked me, ‘do you promise to take malaria treatment weekly until you have completed the full course?’ I promised to take the full course.”—Patient, IDI

Ultimately, these HBM concepts help explain the calculus that patients make in determining primaquine treatment completion. Patients weighed the perceived benefits and perceived barriers: resolving their current medical condition versus the opportunity costs associated with completing their treatment and foregoing work.

## Discussion

This study adds to the literature in exploring the social, structural, and individual determinants for radical cure of *P*. *vivax* framed by the constructs of the Social Cognitive Theory and Health Behavior Model. We provide a deeper understanding about the rational decision-making of mobile, migrant communities and support it with a quantitative data assessment in the same population.

Work and productivity were the main barriers to primaquine adherence and follow up. This study shows the different rationales that incentivize patients to adhere to treatment. On one hand, the fear of productivity loss due to ill health increases adherence [5, 7]. On the other hand, the results suggest the prioritization of work led patients to stop treatment once their condition improved; discontinuing medications for fear of side effects that may jeopardize their ability to work; and forgetting medications due to their work schedules. The nature of migrant work prevented regular follow up for supervision of primaquine treatment. However in some cases, these barriers could be offset by the perceived harm of vivax malaria recurrences (such as having a previous malaria episode) and the benefits of treatment, especially when there was community support such as transport from within a patient’s social network, home visits by health workers, or organizing follow up visits closer to the patient’s home. Additionally, we found that cultural similarities between the clinical team and patients improved the likelihood of primaquine completion. This is likely due to community trust placed in health providers and the health clinic, improved patient understanding and comfort from counseling provided in the patients’ languages, and the importance of cultural norms in motivating patients to adhere to malaria treatment [5, 29, 30, 32, 33].

The findings of this study confirm that work is a significant factor affecting follow up and treatment adherence [5, 6, 11, 18, 43], and also emphasize the role that culture and community play in resolving environmental and behavioral barriers. In line with literature that demonstrates the importance of malaria literacy for follow up and adherence to primaquine treatment [1, 5, 15, 19, 20], our results suggest that cultural cues—such as social norms for seeking and completing malaria treatment—may overcome environmental barriers at odds with patient knowledge. Furthermore, intentionally incorporating behavioral and social mechanisms (educational programs conducted by staff from the same culture as the patients, community-based participatory research [44], SMS treatment reminders [24]) and considering structural support (collaborating with employers, mobile clinics) may improve adherence to primaquine radical cure treatment in migrant settings.

While attention to behavioral and social change are important, in areas such as the GMR where malaria literacy is high, a shorter radical cure regimen for *P*. *vivax* is an additional solution to overcoming low follow up rates and radical cure non-adherence. Single dose tafenoquine addresses the structural barriers in migrant populations who cannot follow up for extended durations. Prescribing tafenoquine requires quantitative G6PD testing, which is increasingly available in *P*. *vivax* endemic areas [4, 34, 45]. Preventing side effects matter greatly to health workers and patients [5, 23, 43].

Using a convergent parallel mixed methods study did not allow for broader explorations into issues reported by only a few participants such as perceived risk and its relationship to increased adherence in patients with a history of any malaria episode. This study may have been limited in its understanding of social and structural determinants as it was performed during the COVID-19 pandemic, which may have created a novel context compared to *P vivax* treatment for this population in the past. Although travel restrictions constrained day 28 follow up, patients could be contacted in other ways to support primaquine adherence. The patients selected for interviews may have been biased towards individuals who were more adherent. Offsetting these limitations, the qualitative data also included healthcare workers and community leaders who provided a wider narrative about primaquine adherence in these mobile migrant populations. In this study, the quantitative measures for “adherence” and “supervision” was complex. The method of confirming primaquine completion was by patient report and may not have been reliable due to problems with self-report as well as social desirability bias. Patients were assumed to be non-adherent if they could not be contacted or visited by day 28, so the measure of adherence to primaquine treatment was confounded by adherence to scheduled appointments. Subjective decisions to supervise treatment were based on risks for adverse effects rather than as a means to ensure drug adherence.

The results from this study inform and affirm our understanding of the factors that influence primaquine adherence in a mobile migratory population setting. Addressing work and productivity barriers, and perceived risks of malaria with culturally appropriate programs is an important means for advancing malaria elimination in hard to reach populations.

## Supporting information

S1 Checklist Inclusivity in global research. (DOCX)

S1 Table Sample quotes analyzed according to the constructs of the Social Cognitive Theory and Health Belief Model as depicted in Fig 2. (DOCX)
