# Supplementary material for: A mixed methods study investigating factors affecting adherence to Plasmodium vivax malaria primaquine radical cure regimens among migrants along the Myanmar-Thailand border
Source: PLOS Glob Public Health. 2025 Jan 16;5(1):e0003615. doi: 10.1371/journal.pgph.0003615 (PMC11737701; doi:10.1371/journal.pgph.0003615)
Supplement: S1 Table — (DOCX) [file pgph.0003615.s002.docx]

## S1 Table. 1. Sample quotes analyzed according to the constructs of the Social Cognitive Theory and Health Belief Model as depicted in Figure 2.

| Quotes | Social Cognitive Theory Constructs / Themes | Health Belief Model Constructs / Themes |
| --- | --- | --- |
| “They do hard work and it [the antimalarial] makes them weaker, so they don’t want to continue anymore…You cannot force and stop those who take primaquine from going back and working while on treatment; they need to stay at home. Even if they are sick and take medication, they will not quit their work.”—Health care worker, FGD  “It is easy to take this drug but usually after two to three days, they don’t want to take this drug anymore because they cannot tolerate the side effects like drowsiness, fatigue, weakness and they worry that if they need to suffer for many days then it will affect their work.”—Health care worker, FGD  “Most of the patients that I know if they feel better, they don’t want to come back because work and money is their priority. For migrant workers, they have to struggle hard so work and money might be their first choice.”—Health care workers, FGD  “They don’t complete the treatment due to dizziness and they do not want to be absent their work.”—Community leader, FGD  “It is important to work daily for migrant workers. They do not go to clinic though they have mild illness They usually go to clinic when they are severely unwell…[Secondly,] they have to move one place to another as they are migrant workers. If their work is finished here they have to move to another place to work. [Thirdly,] they have to follow their boss when he moves to another place. This is the biggest problem.” —Community leader, FGD  “They stay together with their boss. Most migrant workers say they have dizziness and cannot work if they take malaria drugs. Their main purpose is to earn money to repay for their debts or to build new houses for themselves. The main reason is a money issue. It is the most difficult problem.”—Community leader, FGD | Behavioral Factors / Productivity | Perceived barriers / Productivity  Modifying factors / Socioeconomic status  Perceived severity (to adverse effect, financial loss or gain) and cues to action / Productivity |
| “Even for us [health care workers], it’s really hard to urge and persuade them to come back once a day for treatment. It is more difficult especially during the seasonal working period; after they have finished work they are very tired and the drug makes it more difficult for them.” Health care worker, FGD  “It’s a problem, he is a soldier, needs to move and live in a jungle, sometimes he forgets to take medication. Most of the *Plasmodium vivax* patients are soldiers. Most of the relapse cases are because they forgot and didn’t take complete treatment.”—Health care worker, FGD  “I forgot to take malaria drugs for two days while I was breeding cattle to earn money. I forgot to bring drugs with me.”—patient, IDI  “I told the health care workers that I had to work. I had limited time to come here. It was far to come to the SMRU clinic.”—Patient, IDI  “I am afraid of suffering malaria again. I don’t like to be unwell. I want to be cured completely. I cannot work if I am unhealthy. So I took it regularly till the treatment was finished.”—Patient, IDI  Q: Have you ever seen anyone who feel dizzy and vomit among your friends during the treatment? Do they finish the treatment?  A: Yes. They did not listen to the instructions and did not finish the course. They threw the drugs away.  Q: Why did they throw the drugs away?  A: They cannot tolerate the side effects. They feel dizzy and cannot walk and cannot do daily work. As for me, I finished all drugs. I did not feel any side effects.  Q: What will you do if there are side effects for you?  A: I will finish the course though there are side effects for me. I am afraid of dying of malaria. I finished the course completely. I followed the instructions well.—Exchange with patient, IDI  “They never admit they do not finish the [primaquine] course. But we know. Some patients complain of vomiting after they take two doses. They complain they cannot tolerate any more. I explain they have to finish the course otherwise malaria cannot be cured. Some patients are not willing to continue the treatment. Other patients finish the course.” —Community leader, FGD | Behavioral factors / Work  Behavioral factors / Malaria literacy | Perceived barriers / Work  Modifying factors / Socioeconomic status  Perceived severity (to acute malaria and adverse effects) and cues to action / Malaria literacy and Work |
| “Usually, when we test the patient and the result is vivax malaria we explain to them that ‘chloroquine can only clear the malaria parasites in your blood but not inside your liver. We will treat you with primaquine; not everyone can take it. Before we give you this medicine we need to check if you have G6PD deficiency.’…If the result is normal [i.e., no G6PD deficiency] then we explain to them that they have to finish all the medication as prescribed so that it will kill all the malaria parasites in their liver…We explain about the primaquine side effects, all medication has their side effects. If they feel like weak, discomfort, breathing difficulties, abdominal pain, coke color urine, they need to come back to us.—Health care worker, FGD  Q: What did the health care worker tell you about malaria treatment?  A: They said it might make me dizzy. But that it is not a problem. [They told me that] I should stay at home. I had to take black pills [primaquine] after meals. If you take before meals, you may have abdominal pain. I always took after meals.  Q: Did they explain how to take the drugs and side effects?  A: They said that I could get vomiting after taking drugs. But I did not vomit from it…They told me that malaria would be cured after taking three days of treatment. If I took malaria drugs weekly, I would be completely cleared of malaria. So, I tried to take the full malaria treatment.—Exchange with patient, IDI  Q: Did they explain the medicine to you?  A: Yes, they told me how to take the medicine, how many days and the time to take it, and that it must be completed.  Q: Did you take it regularly?  A: Yes, I did…They informed me to come back if I forgot how to take it or if I have any problems at home.  Q: How many days did you take it?  A: 14 days. The first one is once per day for 3 days and the second one is once per day for 14 days. Three times per day for the other one. I must take the medicine regularly otherwise it [malaria] will come back. So I took it all.—Exchange with patient, IDI  “When they suffer dizziness after taking chloroquine, they take boiled traditional medicine leaves. They believe in it. But it is not sure if the disease is cured or not. They tell each other not to be afraid of malaria. They think malaria disease is as usual. Malaria [vivax] does not give them trouble. It is my experience.”—Community leader, FGD | Cognitive factors / Health education  Cognitive factors / Social and community norms | Perceived susceptibility (to recurrences or adverse effects) and cues to action / Health education and Malaria literacy  Perceived severity (to acute malaria and adverse effects) and cues to action / Health education, Malaria literacy, Social and community norms, drug administration |
| “A long time ago, my son was very sick with malaria and he received only chloroquine not primaquine. He suffered from malaria sickness again and again after that. But when he received the primaquine 14 days treatment from SMRU, he never suffers from malaria anymore. The medicine is a bit strong so my son was pale a bit for a few days but never had malaria after that. Malaria comes back because it hides in the liver.”—Health care worker, FGD  “I understood that was malaria because of the headache and body pain. I have to take medicine regularly until it is finished otherwise it would be recurrent.”—Patient, IDI | Cognitive factors / Malaria literacy | Perceived severity (to acute malaria) and cues to action / Malaria literacy  Perceived susceptibility (to recurrences) and cues to action / Malaria literacy |
| “My elder brother went to the SMRU clinic. So my mother asked me to follow my elder brother to clinic the next day…My neighbors had gotten malaria. They came to the SMRU clinic to get malaria treatment.”—Patient, IDI  “My husband asked me to come to the SMRU clinic for malaria. Malaria is not cured by taking traditional medicine. On the Thai side, nobody takes traditional medicine for malaria. They usually go to the clinic for malaria treatment. My friends told me about the clinic. My aunty works here. My aunty told me to come here whenever I have a health problem. I have no Thai documents. I always come here. I see everybody coming to this clinic when they are unwell. Malaria will not be cured if we do not come to the clinic. My friends come to clinic when they got malaria. Malaria is not cured at home.”--Patient, IDI  “There are many unwell soldiers at my site. There are about 50 soldiers. If we are unwell, we usually visit here. We also send our families’ members here if they are unwell after we contact the health care worker.”—Patient, IDI | Cognitive factors / Social and community norms | Perceived severity (to acute malaria) and cues to action / Social and community norms, and access to health services |
| “The first time I had malaria, my husband came back from work to take us to the SMRU clinic. For this [current] episode, I had to bring myself. My husband was working. It took 30 minutes from my house to the clinic by motorbike.”—Patient, IDI  “For those who cannot complete the dose, they are not villagers. They live in a remote place and it is hard for the patient to come back [for follow up].”—Health care worker, FGD  “Yes, we have difficulties in treating that group. Some people live in this village, but they need to go and live in other places because of their duty, business. Some take medication for 2 or 3 days and need to travel, we cannot stop them, we gave them medication and asked them to finish all of it. We are not sure whether they really take it all or not. Even though some people are the villagers, they don’t live in a village, they live on their farm or in the fields and it is not near their village so it’s hard for them to come back and take medication twice a day (in the morning and in the evening). For some families, we asked them to come and supervise their treatment in the morning and give them medicine to take in the evening and come back again in the morning to get more medication.”—Health care worker, FGD | Environmental factors / Access to health services | Modifying factors / Socioeconomic status, mobility, and access to health services |
